# Genetic landscape of breast cancer subtypes following radiation therapy: insights from comprehensive profiling

**DOI:** 10.3389/fonc.2024.1291509

**Published:** 2024-02-06

**Authors:** Fang Wang, Weiyan Wang, Minglei Wang, Dawei Chen

**Affiliations:** ^1^ School of Clinical Medicine, Shandong Second Medical University, Weifang, China; ^2^ Department of Radiation Oncology, Shandong Cancer Hospital and Institute, Jinan, China; ^3^ Department of Hematology, Taian Central Hospital, Taian, China; ^4^ Department of Oncology, Shandong Cancer Hospital and Institute Shandong First Medical University and Shandong Academy of Medical Sciences, Jinan, China

**Keywords:** breast cancer, cutaneous radiation injury, high frequency mutation genes, pathological subtype, Wnt pathway

## Abstract

**Background:**

In breast cancer, in the era of precision cancer therapy, different patterns of genetic mutations dictate different treatments options. However, it is not clear whether the genetic profiling of breast cancer patients undergoing breast-conserving surgery is related to the adverse reactions caused by radiotherapy.

**Methods:**

We collected formalin-fixed paraffin-embedded (FFPE) tumor tissue samples from 54 breast cancer patients treated with radiation after breast-conserving surgery and identified comprehensive molecular information in hundreds of cancer-associated genes by FoundationOne CDx (F1CDx), a next-generation sequencing (NGS)-based assay.

**Results:**

Among our cohort of 54 breast cancer patients, we found high-frequency mutations in cancer-related genes such as TP53 (56%), RAD21 (39%), PIK3CA (35%), ERBB2 (24%), and MYC (22%). Strikingly, we detected that the WNT pathway appears to be a signaling pathway with specific high-frequency mutations in the HER2 subtype. We also compared the mutation frequencies of the two groups of patients with and without cutaneous radiation injury (CRI) after radiotherapy and found that the mutation frequencies of two genes, FGFR1 and KLHL6, were significantly higher in patients with CRI : No subgroup than in those with CRI : Yes.

**Conclusion:**

Different breast cancer subtypes have their own type-specific mutation patterns. FGFR1 and KLHL6 mutations are protective factors for radiation-induced skin toxicity in breast cancer patients.

## Introduction

Breast cancer is the most common malignant tumor in women and one of the three most common cancers in the world (1–3). All cancers have somatic mutations in their genomes, and some of these mutations are associated with tumor development (4). The frequency of breast cancer oncogene mutations is more widely distributed than in other solid tumors and depends on the subtype of breast cancer (5). Genes are pieces of DNA or RNA that carry genetic information (6, 7). A test, FoundationOne CDx (F1CDx), uses DNA extracted from formalin-fixed and paraffin-embedded (FFPE) tumor samples to sequence. Because of the diversity of breast cancer, we used F1CDx to conduct a comprehensive study of its genomic characteristics.

In terms of pathological type, we used immunohistochemistry (IHC) including ER, PR, Her2 and Ki67 to type breast cancer in our study, subcohort: Luminal A-like, Luminal B-like, Her2-enriched and Triple-negative breast cancer (TNBC) (1, 8). In this study we performed a detailed analysis of gene mutation profiles between breast cancer pathological subtypes and it was revealed that different subtypes have different patterns of gene mutation.

Radiotherapy (RT) is a routine treatment for breast cancer patients who have undergone breast-conserving surgery. Radiotherapy removes potential residual tumor cells after breast-conserving surgery, reduces the chances of recurrence and metastasis after surgery, and protects normal tissues at the same time, aiming to improve the outcome of cancer patients and safeguard their quality of life after treatment. But the adverse effects induced by radiotherapy limit the treatment outcome (9). Breast cancer postoperative adjuvant radiotherapy may cause some degree of skin damage, clinically called acute cutaneous radiation injury (CRI). It mostly begins to appear 1-2 weeks after radiation therapy and can manifest as skin pigmentation, follicle expansion, sweat hair loss, erythema, edema, and in severe cases, blistering, rupture, and even infection (10–12). The identification of genetic markers associated with adverse effects may help to develop new programs, and they may be clinically relevant as predictive biomarkers.

## Materials and methods

### Patient inclusion and tissue sampling

Initially, we retrospectively collected 97 breast cancer patients from Shandong Cancer Hospital who received radiotherapy after breast-conserving surgery and extracted baseline demographics and survival data from clinical records. Postoperative FFPE specimens were collected and sent for sectioning and ten unstained 4-5 micron thick sections were provided for each specimen. These sections were then subjected to F1CDx, which was provided by Dean Diagnostics. The study was approved by the Ethical Review Board of Shandong Cancer Hospital and Institute.

### Gene detection

The FFPE tumor tissues of 97 patients were sent to Dean Diagnosis for F1CDx, which is designed to include genes known to be somatically altered in human solid tumors that are validated targets for therapy, either approved or in clinical trials, or that are unambiguous drivers of oncogenesis based on current knowledge. The current assay interrogates 324 genes as well as introns of 36 genes involved in rearrangements. Therefore, it is suitable for detecting mutations at the DNA and RNA levels of specified oncogenes, such as point mutations, small fragment insertion, deletion mutations, copy number variants, and gene fusion variants, and cannot assess mutations and their effects outside the scope of the assay.

F1CDx will be performed exclusively as a laboratory service using DNA extracted from FFPE tumor samples, who is a next-generation sequencing (NGS) based *in vitro* diagnostic device. The proposed assay will employ a single DNA extraction method from routine FFPE biopsy or surgical resection specimens, 50-1000 ng of which will undergo whole-genome shotgun library construction and hybridization-based capture of all coding exons from 309 cancer-related genes, one promoter region, one non-coding (ncRNA), and select intronic regions from 34 commonly rearranged genes, 21 of which also include the coding exons. The assay therefore includes detection of alterations in a total of 324 genes. Assay specifications were determined for typical median exon coverage of approximately 500X. Sequence data will be processed using a customized analysis pipeline designed to accurately detect all classes of genomic alterations, including base substitutions, indels, focal copy number amplifications, homozygous gene deletions, and selected genomic rearrangements (e.g., gene fusions). Additionally, genomic signatures including loss of heterozygosity (LOH), microsatellite instability and tumor mutational burden will be reported. The detection step includes the preliminary pathological quality examination, DNA extraction, library construction, hybridization capture, sequencing, statistical analysis.

A total of 55 samples were successfully tested, and the reasons for failure could be the overtime between sectioning and testing, or the quality of FFPE specimens or possible risk factors during transportation. Our sequencing data files have been uploaded to the National Genomics Data Center (NGDC) under accession code PRJCA019832. All other relevant data and codes used in this paper are available upon request (https://ngdc.cncb.ac.cn/gsa-human/browse/HRA005665).

### Clinical characteristics

Patient’s medical records were assessed, including pathological type, stage, ER, PR, Her2, KI-67, whether postoperative chemotherapy, radiotherapy regimen, whether CRI, grade of injury, whether bone marrow suppression (BMS) occurred, and grade of injury. CRI mostly begins to appear 1-2 weeks after radiation therapy and can manifest as skin pigmentation, follicle expansion, sweat hair loss, erythema, edema, and in severe cases, blistering, rupture, and even infection. BMS is defined according to the CTCAE 5.0 standard.

### Statistical analysis

Since no corresponding clinical data information was found for 1 sample, this sample was removed from the follow-up analysis, and a total of 54 samples were subjected to follow-up analysis. All differences between groups were analyzed by R software, chi-square test, fitted ratio test and Fisher’s exact test. P value less than 0.05 was statistically significant.

## Results

### Cohort

A total of 54 breast cancer samples who all were female were obtained at the Shandong Cancer Hospital and Institute between 2014 and 2019.The clinicopathologic features of the all patients are shown in **Table 1**. There were more patients younger than 60 years (85.2%) and their median age was 45 years (27–76 years). More than 90% of the patients were stage I-II and the rest were stage III. Molecular subtype of breast cancer included Luminal A-like disease (22.2%), Luminal B-like disease (57.4%), Her2-positive disease (7.4%) and TNBC (11.1%). They all had undergone post-breast-conserving radiation therapy and more patients received postoperative chemotherapy (74.1%) than those who did not (25.9%). The radiation doses administrated were below 60 Gy (44.4%) (60Gy included) and higher than 60 Gy (55.6%). More than half of the patients developed CRI (68.5%) and the majority were in degree I (55.6%). Fewer patients developed BMS (37%) than those who did not (63%) and the most were ≤ grade II (31.5%).

**Table 1:** Patients’ clinicopathologic characteristics.

Variable	Classification	Result, n (%)
Age	<60	46 (85.2)
	≥60	8 (14.8)
Sex	Female	54 (100.0)
	Male	0 (0.0)
Stage	I	31 (57.4)
	II	21 (38.9)
	III	2 (3.7)
Subtype	Luminal A	12 (22.2)
	Luminal B	31 (57.4)
	HER2	4 (7.4)
	Triple-negative	6 (11.1)
Type of surgery	Breast-conserving surgery	54 (100.0)
	Others	0 (0.0)
Postoperative adjuvant therapy	Radiotherapy : Yes	54 (100.0)
	Radiotherapy : No	0 (0.0)
	Chemotherapy : Yes	40 (74.1)
	Chemotherapy : No	14 (25.9)
CRI	Yes	37 (68.5)
	No	17 (31.5)
CRI_Grade	I	30 (55.6)
	II	6 (11.1)
	III	1 (1.9)
BMS	Yes	20 (37.0)
	No	34 (63.0)
BMS_Grade	I	4 (7.4)
	II	13 (24.1)
	III	2 (3.7)
	IV	1 (1.9)

The effect of age (p=0.246), tumor stages (p=0.133), molecular subtype (p=0.616) and total dose of radiotherapy (p=0.614) on BMS was not statistical difference. BMS was statistically significant in relation to postoperative chemotherapy or not (p=0.007), but the grade of BMS was irrelevant to chemotherapy or not (**Table 2**). The occurrence of CRI is unrelated to any of the above (**Table 3**).

**Table 2:** Statistical relationship between BMS and clinicopathologic features.

Variable		BMS_Yes N=20	BMS_No N=34	p Value
Age	<60	19	27	.246
	≥60	1	7	
Stage	I-II	18	34	.133
	III	2	0	
Subtype	Luminal A	3	10	.616
	Luminal B	13	18	
	HER2	2	2	
	Triple-negative	2	4	
Total dose of radiotherapy	≤60	8	16	.614
	>60	12	18	
Chemotherapy	Yes	19	21	.007
	No	1	13	

**Table:** 

Variable	BMS_I-II N=17	BMS_III-IV N=3	p Value
Chemotherapy : Yes	16	3	.850
Chemotherapy : No	1	0	

**Table 3:** Statistical relationship between CRI and clinicopathologic features.

Variable		CRI_Yes N=37	CRI_No N=17	p Value
Age	<60	32	14	1.000
	≥60	5	3	
Stage	I-II	36	16	.535
	III	1	1	
Subtype	Luminal A	6	7	.254
	Luminal B	24	7	
	HER2	3	1	
	Triple-negative	4	2	
Total dose of radiotherapy	≤60	15	9	.394
	>60	22	8	
Chemotherapy	Yes	29	11	.465
	No	8	6	

### Overview of genetic variants

**Figure 1:**
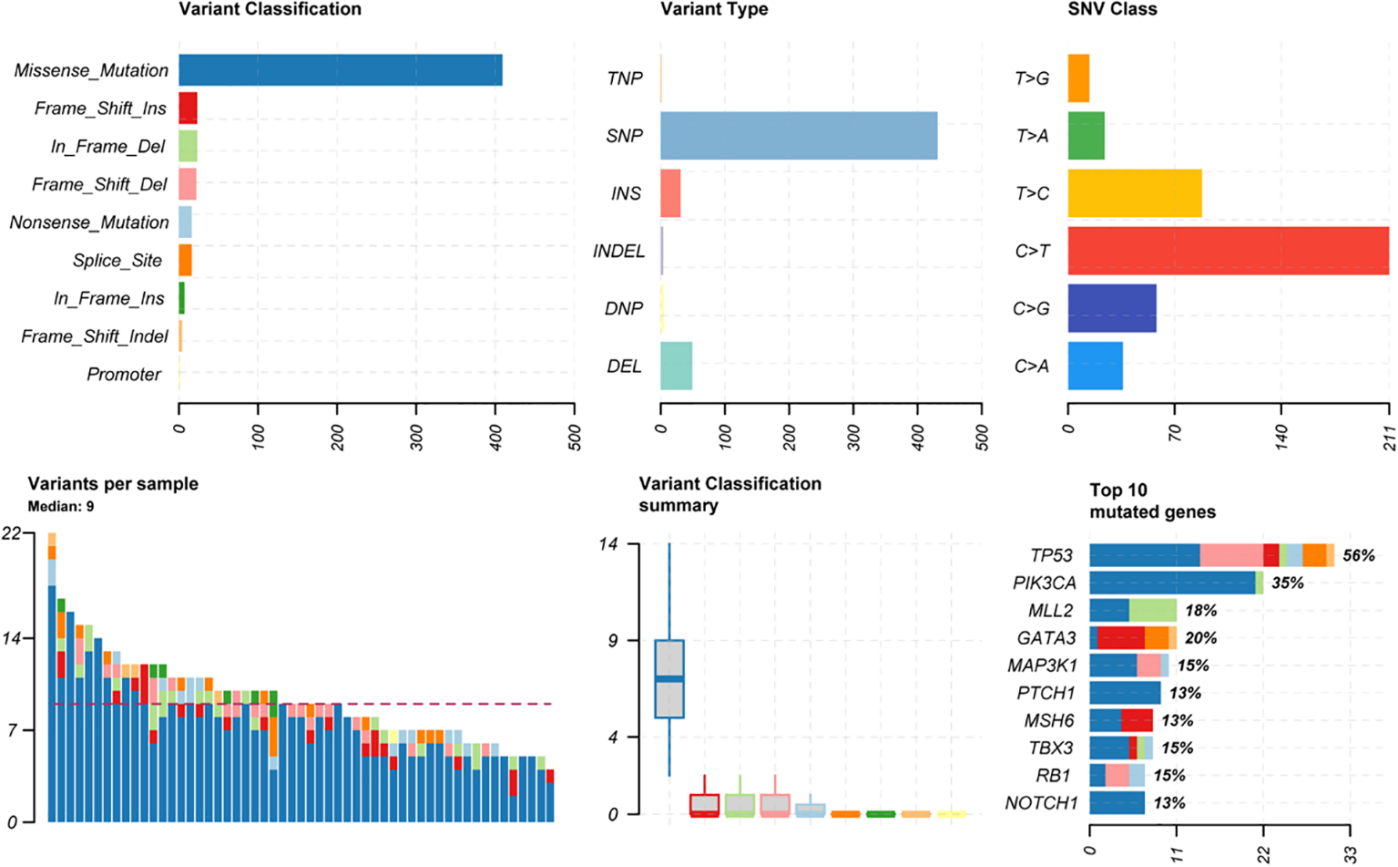
Distribution proportion of SNV mutation types and top10 SNV mutant genes in all samples.

Among the 54 samples, the distribution of genetic variants classificatioin was highest for missense mutations, mostly for SNP types, with the highest proportion of mutations in C > T (similar to most cancer types). The median variants number of patients was 9. The top 5 mutated genes were TP53(56%), PIK3CA (35%), GATA3 (20%), MLL2 (18%), MAP3K1 (15%) (**Figure 1**).

### Gene mutation functional analysis

**Figure 2:**
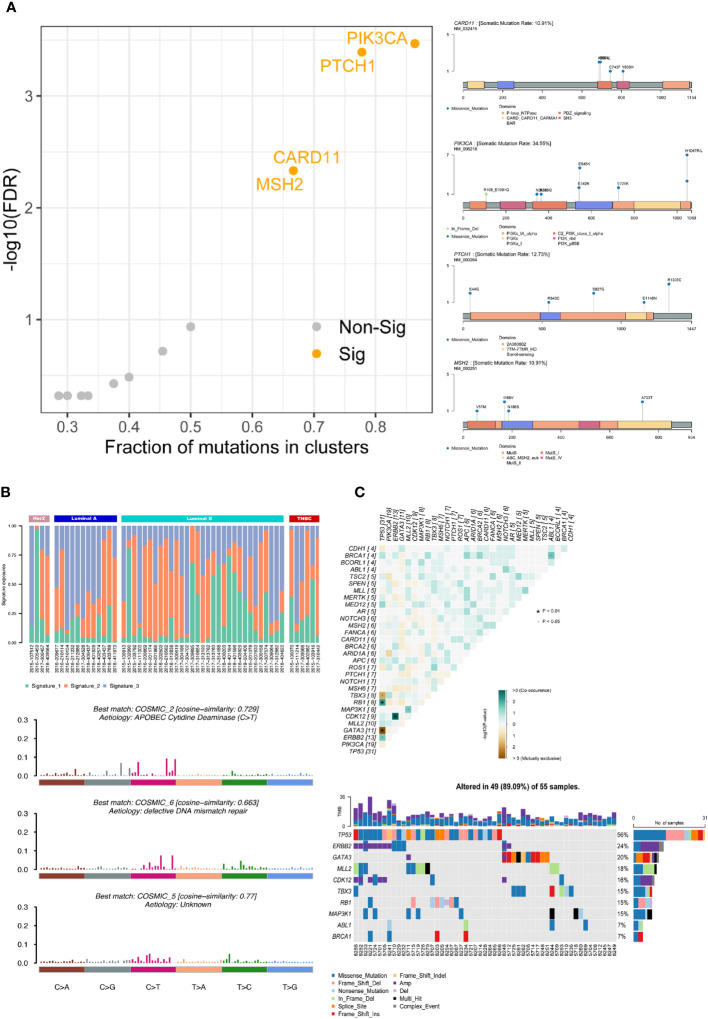
Gene mutation functional analysis. **(A)** Driver gene analysis: calculating the significant driver genes and the specific mutational sites of the four associated driver genes in the samples by the Oncodrive algorithm. **(B)** Mutational signatures analysis: calculating the mutation frequency of this signature in each sample, obtaining the different mutation characteristics by non-negative matrix decomposition, and elucidating the biological significance by comparing with COSMIC database. **(C)** Co-mutant and mutually exclusive mutant gene pairs and specific mutant forms of the above genes in each sample. · P<0.05, * P<0.01.

Using the Oncodrive algorithm, as shown in **Figure 2A**, PIK3CA, PTCH1, CARD11 and MSH2 were calculated to be significantly enriched driver genes. The somatic mutation rate of PIK3CA was 34.55% and there were 8 mutation sites including 7 in_frame_del and 1 missense mutation. The somatic mutation rate of PTCH1 was 12.73% and there were 5 missense mutations. Both MSH2 and CARD11 had a somatic mutation rate of 10.91% with 5 missense mutations. The SNV site and its upstream and downstream bases were formed into triplet base signatures. By calculating the mutation frequency of signature in each sample, 3 significantly enriched mutational signatures were obtained by decomposition using non-negative matrix, which named as signature_1, signature_2, signature_3. The similarity of these 3 signatures with those of known function in the cosmic database shows on the **Figure 2B**. Signature_3, the function of which is unknown, is more prevalent in luminal A-like disease breast cancer and has a similarity of 0.77 with COSMIC_5. Signature_2 is more prevalent in luminal B-like disease breast cancers with a similarity of 0.729 to COSMIC_2, associated with “APOBEC Cytidine Deaminase”. These 3 signatures are evenly distributed in Her2-positive disease and triple-negative breast cancer patients. The signature_1 is similar to COSMIC_6 and associated with “DNA mismatch repair defect”.

Based on the mutation of different gene pairs in the samples, the fisher test was used to conclude that TBX3 and TP53 (p<0.05), GATA3 and TP53 (p<0.01) were mutually exclusive and RB1 and TP53 (p<0.01), CDK12 and ERBB2 (p<0.01), MAP3K1 and MLL2 (p<0.05), BRCA1 and ABL1 (p<0.05), ERBB2 and TP53 (p<0.05) were co-occurrence in all samples. The specific form of mutation in each of the above genes in each sample is shown on the right (**Figure 2C**).

### Distribution of gene mutations in each pathological subtype

**Figure 3:**
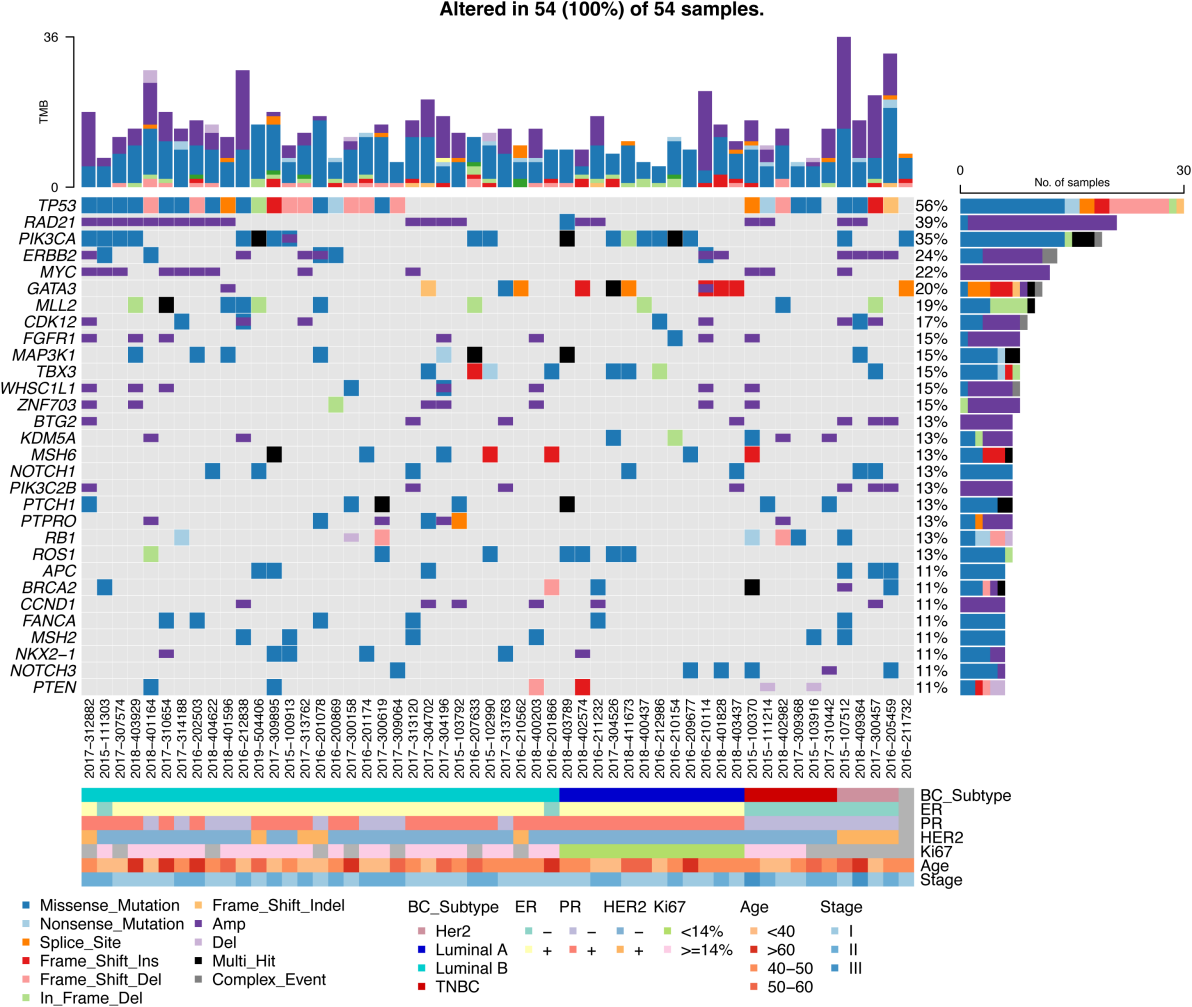
Mutation waterfall plot of all samples.

TP53(56%), RAD21(39%), PIK3CA(35%), ERBB2(24%), and MYC(22%) were the most frequently mutated genes, and TP53 was mutated in 50% of the samples. Most of RAD21 and ERBB2 were copy number amplification mutations, and PIK3CA was a SNV variant. TP53 was mutated at high frequency in Luminal B-like disease, Her2-positive disease and TNBC, but not in Luminal A-like disease. There was no PIK3CA mutation in TNBC in our cohort (**Figure 3**). Overall, different breast cancer subtypes have their own type-specific mutation patterns. We tabulated the mutation frequencies of the top 30 high frequency mutations in breast cancer, grouped by four subtypes in **Table S1**.

**Figure 4:**
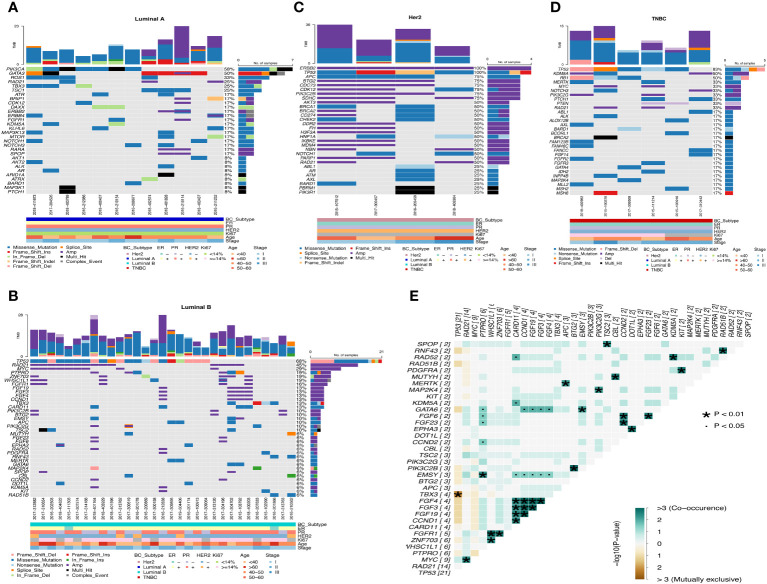
Waterfall map of top30 mutated genes in each subtype of breast cancer. **(A)** Luminal A **(B)** Luminal B **(C)** Her2 **(D)** TNBC **(E)** Co-mutation and mutually exclusive mutation gene pairs of Luminal B subtype. · P<0.05, * P<0.01.

**Figure 5:**
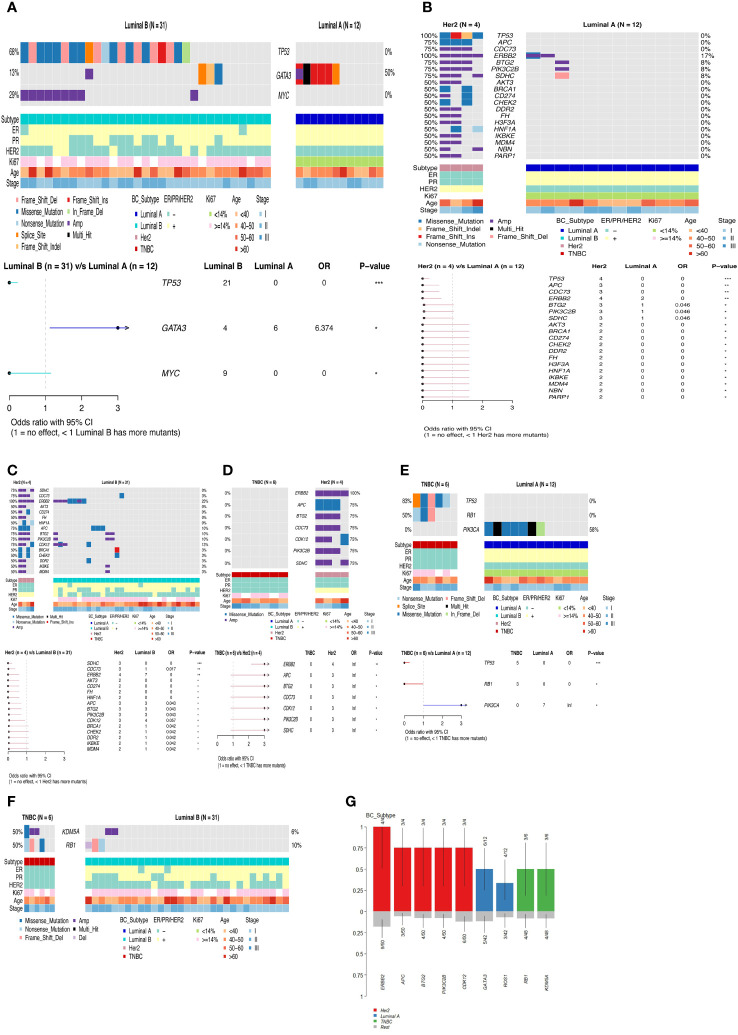
Differences in gene mutation and pathway mutation frequencies among breast cancer subtypes. **(A)** Luminal B vs Luminal A **(B)** Her2 vs Luminal A **(C)** Her2 vs Luminal B **(D)** TNBC vs Her2 **(E)** TNBC vs Luminal A **(F)** TNBC vs Luminal B **(G)** Comparison of clinical enrichment analysis in two subgroups. *P<0.05, **P<0.01, ***P<0.001.

Luminal A-like disease has high frequency mutations in PIK3CA, GATA3, ROS1, RAD21, etc. GATA3 is mostly frame-shift-ins (**Figure 4A**). We found that in two of these cases, although the clinical immunohistochemistry results were Her2-negative, the NGS results showed copy number amplification of ERBB2, indicating heterogeneity at the molecular level in tumors with similar immunohistochemical profiles, and that the standard immunohistochemical markers currently used in clinical practice can well but not fully accurately represent the intrinsic subtype. Luminal B-like disease has common mutations in TP53, RAD21, MYC (**Figure 4B**) and there are co-amplifications of “FGF4/FGF3/FGF19/CCND1”, “MYC/RAD21”, “FGFR1/ZNF703/WHSC1L1”, “CCND2/FGF6/FGF23”, etc (**Figure 4E**). Most of these co-amplified genes are located in close proximity to chromosomes, where so it is likely that copy number amplification of large segments of chromosomes has occurred. Compared to Luminal B-like disease and Luminal A-like disease, there were differences in the mutation frequencies of TP53, GATA3 and MYC genes, with TP53 (68%, p<0.001) and MYC (29%) having significantly higher mutation frequencies in Luminal B-like disease than in Luminal A-like disease. The TP53 pathway (p<0.001) had higher mutation frequencies in Luminal B-like disease, while the PI3K pathway (p<0.05) had higher mutation frequencies in Luminal A-like disease (**Figure 5A**).

Her2-positive disease all had ERBB2 gene copy number amplification and also had mutations in TP53, while BRCA1/BRCA2 also had mutations in half of the patients (**Figure 4C**). Compared with the Luminal A-like disease, the Her2-positive disease has many differentially mutated genes, such as TP53 (100%, p<0.001), APC (75%, p<0.01), CDC73 (75%, p<0.01), and the mutation frequency of the TP53 (p<0.01) and WNT (p<0.05) pathway is significantly different (**Figure 5B**). There are still many differentially mutated genes compared to the Luminal B-like disease, such as SDHC (75%, p<0.001), CDC73 (75%, p<0.01), AKT3 (50%, p<0.05) (**Figure 5C**) and also many differentially mutated genes compared with the TNBC subtype, such as APC (75%), CDC73 (75%)(**Figure 5D**). Her2 subtype also showed significant differences in the frequency of mutations in the WNT pathway compared to the Luminal B-like and TNBC subtypes(p<0.05). These results suggest that the WNT pathway appears to be a signal pathway for the specific high-frequency mutations of the Her2-positive disease.

TNBC patients had high frequency mutations in TP53, KDM5A, RB1, MERTK, MYC, NOTCH3 and PIK3C2G. One patient had only one BRCA2 mutation that was Multi_Hit (**Figure 4D**). Mutations in the TP53 (83%, p<0.001) and RB1(50%, p<0.05) genes were significantly more frequent in the TNBC than in the Luminal A-like disease, while mutations in PIK3CA (58%, p<0.05) were less frequent than in the Luminal A-like disease. Therefore, the mutation frequency of TP53 pathway(p<0.01) in TNBC is significantly higher than that in Luminal A-like disease (**Figure 5E**). Finally, KDM5A (50%, p<0.05) and RB1 (50%, p<0.05) were the most frequently mutated genes in TNBC compared with Luminal B-like disease (**Figure 5F**).

Overall, the mutation frequencies of ERBB2, APC, BTG2, PIK3C2B, CDK12 were higher in Her2-positive disease compared with all other subtypes, GATA3 and ROS1 were higher in Luminal A-like disease compared with all other subtypes, and RB1 and KDM5A were higher in TNBC compared with other subtypes (**Figure 5G**).

### Identification of genetic variants associated with adverse reactions

**Figure 6:**
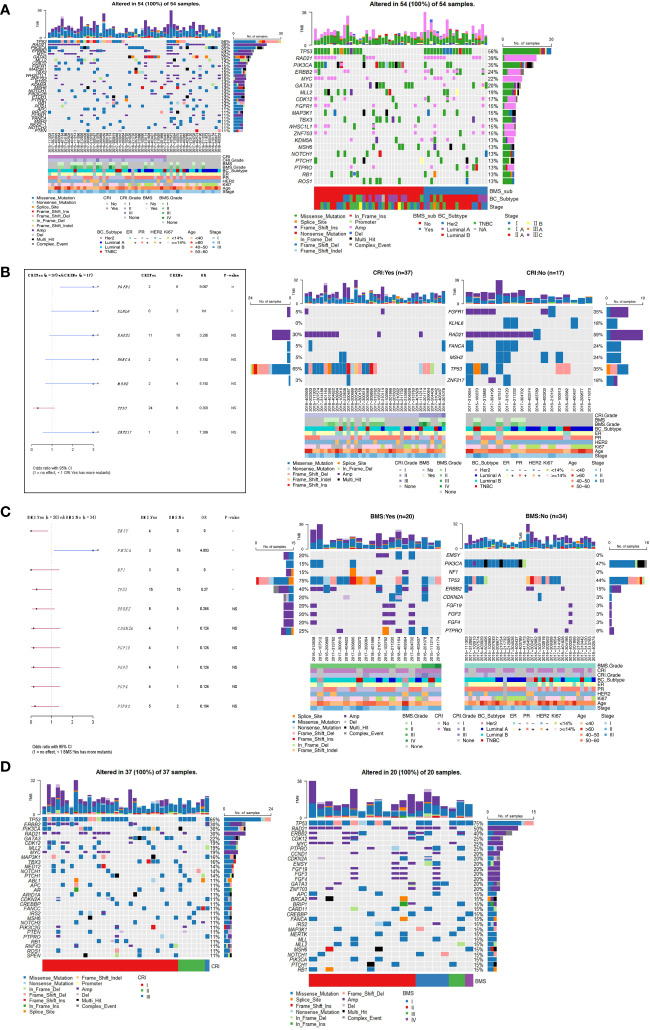
**(A)** Panorama of top30 mutated genes in samples with CRI and BMS. **(B)** Comparison of gene mutation frequencies in subgroups of patients with and without CRI. **(C)** Differences of gene mutation frequencies in subgroups of patients with and without BMS. **(D)** Waterfall map of top30 gene mutations in CRI : Yes group and BMS : Yes group. *P<0.05, **P<0.01. NS, no significance.

The 54 samples were divided into two groups according to whether they developed CRI after radiotherapy. The first group was 37 patients with CRI and the second group was 17 patients without CRI. There were 20 patients with BMS and 34 patients without BMS according to whether they developed BMS after radiotherapy. The top30 mutant gene panorama of 54 cases ranked by CRI with or without mutations is shown in **Figure 6A**, and it appears that the grouped cases of CRI: No and BMS: Yes have a somewhat larger number of mutations. The frequency of FGFR1 and KLHL6 mutations in patients with CRI: No was significantly higher than that in patients with CRI: Yes. The majority of FGFR1 was copy number amplification mutation, whereas KLHL6 was missense mutation only in CRI: No patients (**Figure 6B**). EMSY, NF1 and TP53 were significantly more frequently mutated in the BMS : Yes subgroup of patients than in the BMS : No patients, and it was seen that EMSY and NF1 were only mutated in the BMS : Yes patients, while PIK3CA was more frequently mutated in the BMS : No patients (**Figure 6C**). Specifically, genes with higher mutation frequency in the CRI : Yes Subgroup were TP53(65%), ERBB2(30%), PIK3CA (30%), RAD21(30%). The high-frequency mutated genes in the BMS: Yes subgroup were TP53(75%), RAD21(50%), ERBB2(40%)(**Figure 6D**).

## Discussion

There are few detailed reports on the genetic alterations recognized by the use of F1CDx and their association with clinical molecular subtypes of breast cancer and adverse effects after radiotherapy after breast-conserving surgery. In the present study, we used F1CDx to observe gene mutation profiles in a cohort of patients with various molecular subtypes of breast cancer and whether adverse effects develop after radiotherapy after breast-conserving surgery, which may be clinically relevant.

Several studies have shown high frequency mutated genes in breast cancer as a whole. For example, C. Eric Freitag et al. reported genetic insights into the biology of breast cancer and summarized that the most common clinically actionable genetic alterations identified using F1CDx in a cohort of 223 advanced breast cancers were TP53 (53.8%), PIK3CA (35%), MYC (22%) CCND1 (19.7%) and FGF19 (19.7%) (13). We further mapped TCGA mutation cascades on sangerbox platform, and the database contained a total of 985 samples with detected mutations, of which the mapping samples contained a total of 768(78.0%), and the results showed that the first five mutations were TP53(44%), PIK3CA (42.2%), TTN (24.7%), CDH1(18%) and GATA3(17.1%). These is consistent with the results in our cohort. However, more detailed comparison of alternating differences between races or regions is needed.

Her2 (ERBB2), a member of the Her family of tyrosine kinase receptors (Her1-4), is a major driver of tumor growth in 20% of breast cancers (14, 15). Her2 positivity is a negative prognostic factor for breast cancer patients (16), and trastuzumab, the first approved anti-Her2 monoclonal antibody, is the most commonly used standard of care regimen for Her2-positive breast cancer patients worldwide. The combination of Anthracycline-taxane and Trastuzumab has a higher disease-free survival rate, but the cardiotoxicity caused by anthracyclines is often progressive and irreversible, and because of the reduced cardiotoxicity of docetaxel + carboplatin + trastuzumab (TCH), the TCH regimen is particularly suitable for patients with cardiac comorbidities (16). The chemotherapy regimens of three Her2-enriched breast cancer patients we reviewed exemplified this. Moreover, the WNT pathway appears to be a signaling pathway for Her2 subtype-specific high-frequency mutations by our data results, but there are no specific WNT-targeted therapies available. Our genetic results show that CDK12 is a major oncogenic driver in Her2-positive breast cancer and it is located at chr17q12, exhibiting high concurrent amplification along with Her2. The WNT ligands WNT1 and WNT3 are involved in the activation of WNT/β-catenin/TCF signaling to promote mammary tumorigenesis, and stem cell-like properties in breast cancer (17–21). Hee-Joo Choi et al. found that CDK12-mediated increase in WNT1 and WNT3 expression affects the activity of the typical WNT signaling pathway in Her2+ breast cancer. Alterations in CDK12 expression were accompanied by alterations in WNT1 and WNT3 expression (22). Therefore, CDK12 promotes CSC self-renewal and metastasis by enhancing WNT/β-catenin/TCF signaling, and CDK12 is an actionable target to replace or enhance existing anti-Her2 therapy. And it has also been found that the WNT pathway inhibitor pyridoxal diphosphate (PP) may inhibit BCSC activity by attenuating WNT pathway activity and down-regulating stemness regulators (23).

Our study concluded that mutations in two genes, FGFR1 and KLHL6, are inversely associated with the development of acute radiation-induced skin lesions, this means that patients with these mutations are more likely to be protected from acute radiation-induced skin damage. We found that in some other studies, certain genes have also been elucidated to be associated with radiation-induced skin adverse effects, which will be of reference value in future radiation genomic studies. Sarah Cargnin et al. found that TP53 rs1042522 was associated with the risk of radiation-induced late skin toxicity (24). Eunkyung Lee identified an association between nine SNPs in ATM, CHEK1, RAD51C, TGFB1, and ERCC2 and early skin adverse effects induced by RT (25). Kamalesh Dattaram Mumbrekar describes the sensitivity of mutations in the CD44 and MAT1A genes to acute skin reactions in breast cancer patients undergoing radiotherapy (26).

In terms of potential treatment strategies, we found that there are several targeted therapies available to address genomic changes in FGFR1. Tumors with alterations that activate FGFR1 may be sensitive to FGFR family inhibitors. In addition to the pan-FGFR inhibitor erdafitinib (27), other FGFR inhibitors such as infigratinib, AZD4547, Debio 1347, TAS-120 and the multikinase inhibitors lenvatinib and lucitanib, are under clinical investigation. In a Phase 1/2a study of patients with breast carcinoma harboring an amplification of FGFR1, FGF3, FGF4, or FGF19, lucitanib resulted in a disease control rate (DCR) of 100%; 50% (6/12) of patients achieved PR and 50% (6/12) of patients had SD.

In the present study, we were in an exploratory cohort of patients with breast cancer to identify common genetic variants associated with the risk of radiation-induced acute skin adverse effects. Although some genes were identified by F1CDx as exhibiting nominal levels of significance, this result did not pass multiple corrections and may have been influenced by insufficient sample size. In spite of these negative factors, this study provides some clues for future radiological genomic studies. Subsequently, we will attempt to conduct further studies using a larger cohort of patients with breast cancer to elucidate the association of FGFR1 and KLHL6 or other novel gene mutations with acute radiation skin reactions and consider the potential confounding effects of clinical factors.

## Data availability statement

The datasets presented in this study can be found in online repositories. The names of the repository/repositories and accession number(s) can be found in the article/**Supplementary Material**.

## Ethics statement

The studies involving humans were approved by the ethics committee of Shandong Cancer Hospital and Institute. The studies were conducted in accordance with the local legislation and institutional requirements. The human samples used in this study were acquired from formalin-fixed paraffin-embedded tumor tissue samples from breast cancer patients. Written informed consent for participation was not required from the participants or the participants’ legal guardians/next of kin in accordance with the national legislation and institutional requirements.

## Author contributions

FW: Writing – original draft. MW: Writing – review & editing. DC: Writing – review & editing. WW: Writing – review & editing.
