# Supplementary material for: Genetic landscape of breast cancer subtypes following radiation therapy: insights from comprehensive profiling
Source: Front Oncol. 2024 Feb 6;14:1291509. doi: 10.3389/fonc.2024.1291509 (PMC10878167; doi:10.3389/fonc.2024.1291509)
Supplement: Supplementary file 1 [file Table_1.docx]

| high frequency mutation gene/breast cancer subtype | Luminal A  N=12 | Luminal B  N=31 | Her2  N=4 | TNBC  N=6 |
| --- | --- | --- | --- | --- |
| TP53 | 0 | 68% | 100% | 83% |
| RAD21 | 25% | 45% | 50% | 33% |
| PIK3CA | 58% |  |  | 0 |
| ERBB2 | 17% |  | 100% | 0 |
| MYC | 0 | 29% |  | 33% |
| GATA3 | 50% |  |  |  |
| MLL2 |  |  |  | 17% |
| CDK12 | 17% |  | 75% |  |
| FGFR1 | 17% | 16% |  |  |
| MAP3K1 | 8% |  |  |  |
| TBX3 | 25% | 13% |  |  |
| WHSC1L1 |  | 19% |  |  |
| ZNF703 |  | 19% |  |  |
| BTG2 |  | 10% | 75% |  |
| KDM5A | 17% | 6% |  | 50% |
| MSH6 |  |  |  | 17% |
| NOTCH1 | 17% |  | 50% |  |
| PIK3C2B |  | 10% | 75% |  |
| PTCH1 | 8% |  |  | 33% |
| PTPRO |  | 19% |  |  |
| RB1 |  |  |  | 50% |
| ROS1 | 33% |  |  |  |
| APC |  | 10% | 75% |  |
| BRCA2 |  |  | 50% | 17% |
| CCND1 |  | 13% |  |  |
| FANCA |  |  |  |  |
| MSH2 |  |  |  | 17% |
| NKX2-1 |  |  |  |  |
| NOTCH3 | 17% |  |  | 33% |
| PTEN |  |  |  | 33% |

**Table S1.** Mutation frequency of the top 30 high frequency mutated genes in the four subtypes of 54 breast cancer samples (non-space means that this gene is also a top 30 high frequency mutated gene in the breast cancer subtype).
